# Identifying barriers and facilitators to increase fibre intakes in UK primary school children and exploring the acceptability of intervention components: a UK qualitative study

**DOI:** 10.1017/S1368980024000089

**Published:** 2024-02-01

**Authors:** Angela S Donin, Lucy P Goldsmith, Clare Sharp, Charlotte Wahlich, Peter H Whincup, Michael H Ussher

**Affiliations:** 1 Population Health Research Institute, St George’s, University of London, London SW17 0RE, England, UK; 2 Institute for Social Marketing and Health, University of Stirling, Stirling, Scotland, UK

**Keywords:** School-based interventions, Dietary fibre, Children, Obesity, Healthy diet

## Abstract

**Objective:**

Within the UK, dietary fibre intakes are well below recommended intakes and associated with increased risk of obesity. This study aimed to explore the views of parents and children on barriers and facilitators to increasing fibre intakes and improving diets, alongside investigating the appropriateness of intervention components to overcome modifiable barriers.

**Design:**

Qualitative study including semi-structured interviews and focus groups informed by the Theoretical Domains Framework (TDF) and the Capability-Opportunity-Motivation-Behaviour (COM-B) model.

**Participants:**

Year 5 children (aged 9–10-years) and parents, recruited through London primary schools

**Results:**

A total of twenty-four participants (eleven parents and thirteen children) took part. Five key themes were identified as barriers and facilitators, namely lack of (and improving) knowledge, social factors (including parent–child conflicts, limited time for food preparation, influence of peer and family members), current eating habits, influence of the school, community and home environment in shaping eating behaviours, and the importance of choice and variety in finding foods that are healthy and tasty. Parents strongly supported school-based dietary interventions to enable consistent messaging at home and school and help support dietary behaviour change. Practical sessions (such as workshops to strengthen knowledge, taste tests and food swap ideas) were supported by parents and children.

**Conclusions:**

By using a theory-driven approach to explore the barriers and facilitators to increasing fibre intake, this research identified important themes and modifiable barriers to behaviour change and identifies acceptable intervention components to overcome barriers and bring about sustained dietary behaviour change in primary school children.

Prevalence of overweight and obesity in childhood is extremely high in the UK, the most recent data indicate that during the COVID-19 pandemic childhood obesity increased substantially with nearly 24 % of Year 6 primary school children estimated to be obese^(1)^. Data from the UK National Child Measurement Programme also highlight the dramatic increases (about 12 %) in obesity prevalence from Reception years (aged 4–5 years) to Year 6 (aged 10–11 years), indicating the importance of targeting primary schools with prevention strategies. The prevalence of childhood obesity is also strongly associated with deprivation, obesity prevalence in the most deprived areas being almost twice as high as the least deprived^(1)^. This gap is widening and, along with the ambitious aim of halving childhood obesity by 2030, government strategies also include aims to significantly reduce the gap (childhood obesity: a plan for action^(2)^). This will require dietary interventions which are aimed at more deprived areas and are shown to be acceptable across different socio-economic groups.

Diet plays an important role in obesity prevention. Observational and experimental evidence have demonstrated the protective effects of diets which are high in fibre for obesity prevention (and other diseases including CHD and type 2 diabetes) in adults^(3–5)^ and in children using large-scale prospective data^(6,7)^. Childhood diets low in fibre intakes are associated with increased risk of childhood obesity (about 21 %) compared with high-fibre diets^(8–10)^. Findings from a recent systematic review concluded that amongst well-conducted prospective studies in children, higher intakes of dietary fibre were associated with a healthier weight, improved blood pressure, blood lipids and glycaemia^(11)^. It is also evident that fibre from a range of sources (including fruit, vegetables and cereals) may offer beneficial effects on weight status in children, along with other health benefits^(12)^, though no trials have yet been carried out in children to formally examine these^(11)^.

Previous UK dietary interventions in children, which have generally focused on increasing fruit and vegetable intakes, have shown little effect on changing diets and improving dietary fibre intakes^(13)^. However, a short-term intervention which provided high-fibre breakfast cereals for a 1-month period significantly increased fibre intakes in 9–10-year-old children, demonstrating feasibility and acceptability of providing children with high-fibre food options as a method to increase intakes^(14)^. Within the UK, the National Fruit and Vegetable scheme provides a free piece of fruit or vegetable to every Key stage 1 child (aged 4–6 years) each school day, and this scheme is effective in increasing fruit and vegetable consumption during those years, but consumption then drops during Key stage 2 when children are no longer eligible^(15)^. A process evaluation of an intervention which aimed to sustain the increase in consumption reported a lack of engagement and adherence with the intervention activities by participants, possibly due to the lack of acceptability for the target population^(16)^. Further research is needed to explore the most effective methods to sustain dietary change once food provision is stopped. Dietary fibre intakes in the UK on average are well below recommended intakes, with only about 10 % of adults and children meeting current recommendations^(17)^. Obesity prevention strategies which focus on increasing fibre intakes in children are therefore relevant at a population level and could improve weight maintenance alongside many other important health outcomes^(11)^.

Disappointingly, obesity prevention programmes which have attempted to improve diets tend to show little change on weight-based outcomes^(18)^, particularly in older children^(19)^. Several reasons have been suggested to explain this lack of effect, including inappropriate study duration, non-targeted population-wide interventions, use of BMI as a main outcome of body composition and, perhaps most importantly, a lack of psychological theory to understand the mechanisms of the targeted behaviour change^(20)^. Furthermore, the Medical Research Council/National Institute for Health and Care Research framework for developing complex interventions includes the development phase of the intervention and stresses the importance of including diverse stakeholder perspectives and an evaluation of the context which the intervention will interact within^(21)^. The aims of the current qualitative study are therefore to explore the barriers and facilitators towards adopting high-fibre diets in primary school aged children using focus groups and interviews with both parent and child groups and to explore the acceptability of intervention elements which might overcome barriers.

## Methods

We used a qualitative, descriptive methodology, conducting semi-structured focus groups and interviews with parents of Year 5 primary school children and semi-structured focus groups with Year 5 primary school children. Year 5 was chosen as an appropriate age for this methodology^(22)^, in which the influence of dietary factors on adiposity has been shown^(23)^. The inclusion of group activities, shorter sessions and simpler language was adopted for the child focus groups to help engagement. A social constructivist theoretical paradigm was used in our approach to this study, recognising the creation of attitudes and behaviours through interaction with others.

### Recruitment

Schools were purposively sampled from two areas of London, and this method of sampling was used in order to recruit schools in areas with both ethnic and sociodemographic diversity: Tooting in Wandsworth and Walthamstow in Waltham Forest. Four out of five schools approached agreed to take part (one subsequently dropped out due to staff illness). All children, and parents of children, in Year 5 classes in the remaining three participating schools were invited to take part in the study. The researchers visited the schools and discussed the research with the Year 5 classes, and participant information sheets (PIS) were then sent home explaining the purpose of the study. Instructions were included for parents to complete and sign consent forms if they and/or their child would like to take part. Parent participants received a £20 gift voucher as a thank you for their time.

### Parent interviews and child focus groups

All interviews and focus groups were conducted within the school setting, in quiet, private rooms. The gender balance was considered in each school when forming the child focus groups, and where possible the parent interviews. Before beginning the interviews/focus groups, the interviewers (ASD and LG) introduced themselves and briefly described their background and involvement in the project. ASD is a Lecturer in Epidemiology (PhD) and has the experience of conducting qualitative research with children, and LG is a senior postdoctoral researcher (PhD), who has extensive experience in conducting a range of qualitative research in adults and children. They used a topic/question guide while allowing participants to speak freely. The topic guides for parents and children are included as Supplementary Tables 1 and 2. The topic guide was informed by the Theoretical Domains Framework (TDF) and the Capability-Opportunity-Motivation-Behaviour (COM-B) model in order to explore a broad range of potential barriers and facilitators influencing dietary change in children. The TDF defines fourteen domains of determinants of health behaviour (e.g. social influences, beliefs about consequences of behaviours, cognitive and interpersonal skills and goals) and is commonly used in qualitative work to guide questioning around barriers and facilitators to behaviour change^(24)^. We used the COM-B model^(25)^ to prompt questions about barriers and facilitators, specifically, according to whether they are related to the: (i) capability to enact the required behaviours (e.g. having adequate knowledge about diet), (ii) opportunity to engage in the behaviour (e.g. having appropriate advice) and (iii) motivation towards the behaviour (e.g. level of willingness to change diet). The interviews and focus groups lasted about 45 min and were audio-recorded (Sony MP3 IC Recorders) and transcribed (no further field notes were made). Transcripts were not returned to participants at any time. Data saturation was determined when no new themes were reported in the data, and this was confirmed by two researchers.

### Analysis

Thematic analysis^(26)^ of the data was both deductive, informed by TDF and topic guide, and inductive, from participants’ accounts. Using NVivo12, two researchers independently coded 20 % of randomly selected transcripts, for both parents and children, and agreed a coding framework. Two researchers then independently coded all the transcripts. Using an iterative approach, coded themes were used as the categories for analysis and were refined, interpreted, labelled and discussed with the wider team until consensus was reached. To indicate the frequency with which themes were provided by participants, we use the terms ‘all’, ‘almost all’, ‘most’, ‘some’ and ‘a few’. The results are reported according to the consolidated criteria for reporting qualitative research tool (COREQ)^(27)^.

## Results

In total, eighteen parents and eighteen children consented to take part, eleven of the eighteen parents were present on the day and participated and thirteen children participated in the study. It was felt that with this number of participants data saturation was achieved, our approach to determining data saturation involved examining the frequency of new themes emerging following an initial review of the data from two parent interviews and two child interviews^(28)^. Using the coding framework developed during the initial review to determine if new themes or subthemes were emerging, data saturation was agreed when no new themes were needing to be coded, and this followed a review of the remaining two child focus groups (*n* 6) and four parent focus groups (*n* 8) resulting in no further focus groups being arranged. The adult participants were all parents of children in Year 5 primary school (aged 9–10 years), with ten females and one male participant, from diverse ethnic backgrounds (five parents of South Asian, five of white European and one of Eastern Asian heritage). Due to insufficient numbers of parents being available at one time, rather than conducting a focus group, we conducted both dyadic parent interviews (five interviews) and an individual interview (one individual). Child participants were all in Year 5, aged 9–10 years (70 % girls). We conducted four focus groups, three of these included three children, with the remaining group including four children. All interviews and focus groups were conducted in schools during July 2022.

We identified themes from the interviews and focus groups under two headings, corresponding to the aims of the study: (1) children’s and parent’s perceived barriers and facilitators to increasing fibre intakes, and (2) children’s and parent’s views on acceptable intervention characteristics.

### Themes related to barriers and facilitators

Five themes were identified related to perceived barriers and facilitators to increasing fibre intakes, namely knowledge, social factors, habit formation, environmental factors, choice and the need for variety. The school names have been coded below (BW, FD and HM) and not named to maintain anonymity.

#### Knowledge about fibre and about health benefits

Some parents reported limited knowledge or awareness about fibre and its importance for health, stating that they had more awareness of the need to reduce dietary sugar and fat. *‘Yeah, the emphasis is always on fat and sugar and it’s never on fibre actually.’* (R1, BW Parent 2). One parent commented that it is difficult to know which snacks are healthy *‘My daughter says, can I have a healthy snack for school, and so you think oh god, what’s healthy.’* (R1, BW Parent 2); others felt that it would be helpful to have information on the importance of fibre and information to help them identify high-fibre snacks when shopping.

*‘… a lot of parents don’t know what the fibre is, and they would think oh, okay, it’s a normal thing. They don’t understand what the importance are? So, probably that would help if they knew the importance of fibre and how can they balance their diet would really help. I think it’s all about them not being aware of it.’* (R1, FD Parent 1)

*‘It would be helpful because sometimes I don’t understand. The only thing I do understand is the traffic light system on the packaging.’* (R1, BW Parent 2)

Some of the child participants were able to list food items that were high in fibre (including brown bread, brown rice, fruits with skin on, and some cereals including Weetabix and muesli). Most children were less knowledgeable about the specific benefits of eating fibre, although one child talked of its link to heart health and that eating fibre was generally good for you ‘*Cause like it slows your heart rate. It’s better for your body and you hopefully live longer.’ (R1, HM Child 1).*

#### Social factors

Social factors were identified as important barriers and facilitators, among both parents and children, in terms of the influences of parents, peers and the wider family.

### Parent/child conflict and choice

When asked how they would feel about changing foods they offered their children to higher fibre food items, almost all parents stated there was sometimes conflict between what they would like to give their child for lunch and what their child wants. To reduce conflict, when buying food items, some parents said they were influenced by what their child likes to eat; ‘*That’s because I know they’ll eat those*.’ (R1, BW Parent 2). One parent, who was concerned about their child’s reluctance to eat a varied diet, described how their child would decide what food items are bought in the supermarket:

*‘I take her shopping, I say, supermarket, here whatever you feel like buying, I’m not going to say no, just put it in the basket, the trolley and we’ll pay for it. I don’t know what to buy. And she wanders round the whole supermarket and in the end, she’ll just pick up what she normally eats.’* (R1, BW Parent 2)

Some parents recognised that their children want to have some element of control or choice, including over food items:

*‘We’d feed them, but now they have their own choices they don’t want to eat it*.’ (R2, BW Parent 2)

*‘Yeah, but then at the same time they’re at that age where they’re testing boundaries and they don’t like to be controlled really. So, from time to time, you know.’* (R1, HM Parent 1)

### Limited time

Some parents reported having adequate time as an important facilitator to increasing fibre intakes and that their limited time for shopping, cooking and thinking about healthy choices might restrict their ability to provide a healthy, high-fibre diet, often opting for faster, low-fibre options instead:

*‘a lot of parents… because they’ve been working the whole week so there’s a lot of take-away coming home as well.’* (R1, FD Parent 1)

### Parental roles/conflicting attitudes

Differing parental attitudes towards healthy eating and levels of engagement in the children’s diet was reported to be an additional barrier:

*‘My husband he comes home late, he comes 8:30, by the time he comes he doesn’t know what the kids have eaten.’* (R2, BW Parent 2)

*‘I’m more healthy than daddy. Whereas daddy will go and get the Haribo selection from Sainsbury’s, whereas I’m a little bit more health conscious than he is. He sees it as stuff that you remember from childhood and the joy and the memory, ’cause that’s what he had, he had the cereals, the naughty cereals and the naughty snacks. And you can connect that as a child with feeling loved and having fun. You know what I mean? The fun aisle in the supermarket.’* (R2, HM Parent 1)

### Influence of wider family

Different family attitudes towards food or dietary routines around diet was seen as a potential barrier by some parents:

*‘Well my mother-in-law, no, definitely not. …even when she’s cooking she puts a lot of oil, five litres gone in a month and I’m like my god, mine takes like five months. No, she wouldn’t change she’s too set up in her ways. I think the older generations no.’* (R2, FD Parent 1)

Some children also mentioned different dietary routines and food choices by wider family members; *‘…when you go to your grandparents house of course you are going to get sugary stuff.’* (R1, HM Child 1).

### Peer influences

The attitudes and behaviours of the children’s peer group were considered to be both potential barriers and facilitators. A potential barrier of increasing fibre intakes was a peer norm of buying sweets or consuming other unhealthy foods:

*‘Yeah, on certain days. So he gets to go to the shop. But it’s going to be a treat, the sweets, walking home with his friends. So I think it’ll be hard to sort of slip in, would you like some breadsticks and a piece of fruit. I don’t think it’s going to go down well.’* (R1, HM Parent 1)

However, peer influence can be a facilitator when the food eaten by peers is a healthier choice. Some parents felt that children’s eating habits can be positively influenced by the dietary behaviours of peers or positive role models, such as older children they look up to.

*‘Yeah hmm. And also, actually children when they see other children eat, they think oh, she’s eating that, I might try some of that. Whereas at home we’re quite isolated’* (R1, BW Parent 2)

Children were said to enjoy and be more likely to do things if a group is involved, for example, one child who ate fruit and veg at camp because the whole group were doing it.

*‘.he ate so much fruit and veg because it was just there on the table and everyone else was eating it. So I think peer pressure was really positive and if all the other kids are doing it.’* (R1, HM Parent 2)

*‘…quite often if [child] wants something it’s because one of her friends has it in their lunch box.’* (R2, HM Parent 2)

Some children, although not all, indicated they would be more inclined to eat the high-fibre foods if their friends or peer group were also doing it *‘It would motivate me’* (R2, BW Child 1) ; for others, they thought it would be influential if people on social media or those considered to be ‘popular’ were also doing this:

*‘Well, I feel like if other people take part, I would do it too because sometimes I don’t really like when I’m the only one who does it.’* (R1, FD Child 2)

#### Habit formation

Some parents felt their child’s current eating habits may act as barriers to dietary change, and this was consistent with comments by some children. This included their liking for unhealthy or sweet snacks, a refusal to eat healthy food, eating only a very limited diet and reluctance to try new foods.

*‘This is always a big challenge because most kids like sweetness.’* (R, BW Parent 1)

*‘It’s quite hard because my daughter doesn’t eat healthy food. She hardly eats anything anyway. My daughter is a really picky eater anyway, so trying to find a healthy snack to fit in with that it’s so hard.’* (R1, BW Parent 2)

Certain time points were viewed as particularly challenging in terms of changing eating habits, for example, during weekends and holidays. Almost all parents discussed the difficulties of avoiding sweets and unhealthy snacks after school as this is, habitually, what their children wanted.

*‘And that post-school time is when they are, kind of, usually just really quite desperate for something, and quite often it’s, like, a sweet hit.’* (R2, FD Parent 2)

Most children also reported that at certain times in the day they would struggle to change their eating habits, for example, breakfast and straight after school.

*‘Probably breakfast because I usually have white toast, white bread. I like brown toast but usually my brother always likes white toast so I’d try and get him to have brown toast.’* (R3, FD Child 1)

*‘Sometimes you just come out of school, and you are really tired and you just need that boost of sugar.’* (R3, HM Child 1)

#### Environmental factors

When discussing the possibility of a school-based intervention to encourage children to consume more high-fibre foods, all parents felt strongly that this would be hugely beneficial for their children and act as a facilitator for parents to help their children increase their fibre intakes. General comments from parents tended to focus on the value of school-based dietary interventions to improve diets in children and the impact that would have on their child’s health and behaviour.

*‘If they’re healthy, then they’re going to perform better in school and they’re physically fit as well. The diet does really help, it plays the main role in the child’s life, anyone’s life, you feel healthy and if you’re eating healthy foods then obviously it does effect…’* (R1, FD Parent 1)

The school setting for interventions was also seen as the best place to introduce and encourage high-fibre food consumption by all parents and children; children were perceived to respond more positively when the message about eating comes from school and parents felt it could lessen the burden on them as their role would be to reinforce the message coming from the school.

*‘Yes, because sometimes kids don’t listen to the parents more than their teacher. If the teacher says something, oh mummy we have to do this.’* (R2, BW Parent 2)

*‘I often find when…[name] was saying today we had someone come into school, we had a presentation on this. And then he’ll bring that home and then you apply it, you know, these are the snacks from this. So then you’ve got that all ready to start and, you know, that sets them up. So we don’t really have to do that much, we don’t have to explain to them that this is healthy eating. It’s like we’re just reinforcing at home, so we’re supporting it, you know?’* (R1, HM Parent 1)

#### Choice and the need for variety

Some children said they would be more inclined to change their diet and increase fibre intakes if they had a variety of healthy options to choose from *‘Probably if there were loads of varieties of it, like different things.’* (R4, FD Child 1). Liking the taste of the foods was also important to children *‘Knowing that it’s good for me won’t do as much as liking the taste.’* (R3, HM Child 1).

### Themes related to children’s and parents’ views on acceptable intervention characteristics

These themes emerged during discussions around a potential school-based dietary intervention which aimed to increase fibre intakes. The intervention was described as asking children and parents to try a range of high-fibre foods and consider adding these in place of low-fibre options eaten at breakfast, school lunch and snacks. Potential intervention activities were discussed which included offering parent information packs and practical advice, vouchers and free samples of high-fibre foods, school-based taste tests and workshops for children, and other motivational fun activities for the children. The intervention was discussed in general, and six themes were identified as important factors to consider and include, namely, the importance of the introduction to and rationale for the intervention, tailoring, social support, behavioural regulation, practical aspects and modes of delivery. The final theme of practical aspects and modes of delivery was a more detailed discussion on parents’ views on particular intervention activities, including those mentioned above.

#### Introduction to and rationale for an intervention

All parents felt it was important to fully introduce any dietary intervention and the rationale behind it. Parents were keen for researchers to supply them with information to help support their child to participate in a study which aimed to improve their diet. This included information on the health benefits of fibre in diets, to enable parents to explain to their children about why they should be eating high-fibre items. Most parents also wanted information about and examples of high-fibre snacks and packed lunch items they can buy. Having these examples were viewed as particularly helpful for busy parents.

*‘Yes, first of all the parents need to know which one is good, then you can teach the kids. The best way is the parents also know these things.’* (R, BW Parent 1)

All parents felt that both taste tests and free samples would be helpful in encouraging their children to try high-fibre foods and in buying the items. A couple of parents (one of whom had a child with allergies) added that they would wish to know what the food items were first.

*‘Yeah, if they obviously enjoy the taste, if they were nutritionally suitable and…I’d do that, yeah, absolutely.’* (R2, FD Parent 2)

*‘.I’d be open to it if I could try them. I’ve never tried them.’* (R2, HM Parent 1)

All children were also very positive about the idea of being able to try the foods first and having taste tests in school with their peers.

*‘Taste tests are really fun.’* (R2, BW child 1)

*‘I just like changing and trying new things.’* (R1, FD Child 1)

*‘I’d prefer if I knew what it was first and then if I liked it, so I can try the stuff and then carry on.’* (R4, FD Child 1)

#### Tailoring of an intervention

Most parents and children felt it would be important for the research team to have some knowledge of the culture, food types and diets relating to different ethnic groups and also highlighted the need for the research to be communicated in different languages in certain areas, including translated written materials for parents.

*‘And the demographic of the school there are lots of people who are from backgrounds from different cultures, from different backgrounds, so I think it would definitely help to be able to incorporate that, to engage with the parents.’* (R1, BW Parent 2)

Some parents felt that starting off with small changes – *‘baby steps’* (R2, HM Parent 1), introduced slowly and progressing according to individual needs would be preferable and more achievable.

*‘I guess as with anything; you integrate it into the day. It might not seem…you know, it doesn’t necessarily need to be a huge change…’* (R2, FD Parent 2)

Many parents suggested regular communication would be useful to remind them of goals, and one parent raised the question of whether this could be two-way so as to indicate if their child was not able to make or sustain dietary behaviour changes. Many children also thought it would be useful to have regular monitoring and feedback to see how well they were doing.

*‘I just think we don’t need rewards, just to know how we’re doing.’* (R1, FD, Child 1)

#### Social support

All parents felt that they would be able to support their child to change their behaviour if they were involved in the research and this involvement would help facilitate uptake of fibre in their children’s diets. Another parent felt that encouraging their child to increase fibre in their diet should be a joint venture between parents and the researchers who developed the intervention. This would allow parents to have a better understanding of what the intervention would be aiming to achieve and why this was important, and some parents suggested a lack of understanding can act as a barrier to an intervention.

*‘.it’s like you have to justify why you’re stopping the child and if the parent himself doesn’t understand…so it’s quite hard’* (R1, FD Parent 1)

*‘I think it’s also important that…the other thing I would say is that the parents have to be the example, the role model. You can’t tell the children, oh you need to be eating that when you’re not eating that, you see.’* (R1, FD Parent 1)

All children felt that their parents would encourage them to eat high-fibre foods, and this encouragement from parents, often mothers in particular, was considered to be helpful. In two-parent households, some parents stressed the importance of involving both parents and felt this to be beneficial in encouraging children to make the dietary change and help reduce conflict.

*‘.if you get the mother and the father or the other parent involved, it engages them too rather than them just being like a passive kind of oh, why do we have to do this?’* (R2, HM Parent 2)

Parents felt that being able to connect with other parents to share information, advice and tips would be helpful for them. There was interest in online chat groups for parents which meant parents could get involved in their own time.

*‘I wouldn’t mind having some recipes from other mums and say, how about you try making this because it’s really good.’* (R1, BW Parent 2)

*‘I think for busy parents, forums where we do a chat, you know, is usually always very helpful.’* (R1, HM Parent 1)

#### Behavioural regulation skills for children to resist unhealthy snacks

Children talked of things they would do to try to resist eating unhealthy snacks and the beneficial emotional consequences if they managed to achieve this. Techniques included self-talk, distraction, behaviour substitution, for example, eating a substitute snack such as fruit, anticipated reward, that is, thinking about when they will be able to next have the sweet treats or rewards and creating graded tasks such as gradually eating less and less of the unhealthy snacks. Children said they would feel ‘proud’ to have achieved the dietary change.

*‘I would eat fruits like grapes, oranges and apples just to distract myself from eating it, the other stuff.’* (R1, FD Child 1)

*‘Well, I would eat most healthy stuff on the weekdays and then maybe some sweet things on the weekend so like I just think, well, I’ll get to eat that on the weekend so I just think that and then be like, okay, so I just have to wait a few more days and then I’ll get it.’* (R1, FD Child 2)

#### Practical aspects of an intervention

Parents were in favour of a simple and focused approach involving small changes, potentially to foods eaten for breakfast, lunch and snack times.

*‘…a list of suggestions that parents could buy themselves. You know, I mean, the free thing is always going to be helpful but maybe it doesn’t always necessarily have to be actual samples, it could just be a recommended list’* (R2, FD Parent 2)

Parents were happy for intervention activities to involve items of their child’s lunch or afternoon snack to be substituted for high-fibre equivalents, citing the benefits for their child’s health or for the whole family.

*‘Super happy with that idea because it’s good for their health. I’m happy if they would do that. These things are good.’* (R2, FD Parent 1)

*‘Yeah, definitely, for the overall family…the health of all the family. That’s the thing, it’s not just the children that are going to benefit.’* (R2, FD Parent 2)

When asked how children would feel about swapping their usual snacks for high-fibre versions, some of the children expressed some reluctance on the basis that they may be asked to eat foods that they do not like.

*‘I would feel…I wouldn’t eat a banana. I don’t like bananas.’* (R3, BW child 1)

However, the children were more accepting when the swaps involved items they liked, for example, certain fruits – *‘I really like grapes’* (R1, BW Child 1).

Children also talked about an initial reluctance to try high-fibre foods but that this would change to acceptance of the new foods.

*‘I guess I’d be pretty annoyed, but I’d get used to it eventually.’* (R, HM Child 1)

Some parents perceived that high-fibre snack items would be more expensive to buy. They welcomed information about how the price of these items compares with snacks that are lower in fibre and felt that they would be more encouraged to buy items if they did not cost more. A few parents also commented that the high-fibre options should be available in the types of shops they already use, for example, mainstream supermarkets rather than having to go to health food shops. Parents also talked about certain supermarkets being generally more affordable.

*‘Also accessible as well, making sure the foods are accessible, because we don’t shop in health food shops or anything. Supermarkets, so as long as those items are available in the mainstream.’* (R1, BW Parent 2)

#### Modes of intervention delivery

Parents were asked for their views on different activities that could be used to help keep children engaged with an intervention, including workshops/meetings with the children, poster competitions, arts and crafts, chart and stickers and rewards. There was support for all of these to some extent, with some mixed views about the usefulness of charts and stickers for some children. Parents were particularly supportive of fun activities that involve the children, including making things, going on trips or playing games.

*‘Games generally are pretty good. Sometimes the charts can…certainly at the ages that they’re at now, they’d probably find them a bit, sort of, babyish perhaps. But games and anything that they can be more involved…yeah, that kind of thing can be helpful.’* (R2, FD Parent 2)

*‘It would be good if you had a plan. I mean, he still loves a plan, he still loves a chart actually, he still loves a chart.’* (R2, HM Parent 1)

Children were keen to do activities, especially where this was a fun activity such as a quiz or video game on a study website or an activity that could be done in a group. Both children and parents identified goal setting with rewards as highly motivating.

*‘Yes, because I feel like I’d want to do it more.’* (R1, FD Child 2, in response to rewards being offered)

Suggestions from children for rewards included badges, a cuddly toy, a certificate with or without a voucher at the end of a study and being able to have a treat at the weekend.

*‘Or maybe like a little…maybe like a note and then it can be like in this thing where it doesn’t rip or something, so it tells you like a nice quote like you’ve done well or something.’* (R1, FD Child 2)

Finally, the identified themes were mapped to the theoretical components of the TDF and COM-B model which informed the topic guides for the questions. Supplementary Tables 3 and 4 present these along with proposed recommendations for intervention design.

## Discussion

This is the first theoretically informed study to explore barriers and facilitators towards adopting high-fibre diets in primary school aged children and to consider potential intervention elements to address these barriers and facilitators. Among both parents and children, several modifiable barriers and facilitators were identified. Parents and children expressed a clear interest in increasing fibre intakes and improving diets, and the school setting was seen as the appropriate and acceptable environment for dietary interventions for children. There was a perceived lack of knowledge of high-fibre foods and parent responses tended to broaden out to discuss experiences relating more generally to healthy foods. There was a lack of confidence in the ability to change their child’s dietary behaviours due to several social and environmental factors, and these included parent/child conflict over food choices, current dietary habits and a resistance to try new foods, lack of time, influence of family and friends and the influence of their school and community environments.

Despite perceived barriers, parents and children were positive about the possibility of improving diets through a school-based dietary intervention and discussed facilitators which they considered important to help achieve dietary behaviour changes. These included activities to increase knowledge about fibre, the positive influence of peers and role models, a strong motivation for children to follow school-led messaging around healthy eating, and the availability of lots of choice and variety of fibre-rich foods. Several specific intervention components were identified to overcome barriers, and these included clear and practical information on how to increase fibre intakes which needed to be culturally appropriate (e.g. simple foods swaps in packed lunches), opportunities to taste foods, free samples and shopping vouchers for high-fibre foods, achievable goals to gradually introduce dietary change, and parental involvement, and support was seen as a key element in a school-based intervention.

### Consistency with previous research

The importance of the school setting for delivering healthy eating interventions is well established in the literature; children, parents and school staff are overwhelmingly in favour of school-based healthy eating messaging and interventions to encourage dietary change^(29–31)^. Children report finding the consistent healthy eating messaging across home and school settings supports them to help them make healthier food choices^(32)^, parents report the benefit of the authoritative messaging from schools to reinforce the changes made at home^(33)^, and school staff also acknowledge the importance of the school setting for providing the opportunity to deliver nutrition education and healthy role modelling^(33)^. A large multi-country, qualitative study exploring parent views on school-based dietary interventions highlighted that both effective communications between school and parents and parental involvement were important to encourage healthier eating habits and dietary changes in both school and home settings^(34)^, findings echoed in our own research and others^(35,36)^. This consistency across studies is perhaps not surprising when we consider the importance of the school setting as a major environmental influence effecting a child’s knowledge and opportunities to make healthy decisions. Consistent health messages and opportunities across schools and homes would increase the impact and effects on health behaviours.

Increasing parental knowledge about fibre-rich foods is clearly a key element for UK interventions which aim to increase fibre intakes. School-based dietary interventions provide the opportunity to not only incorporate nutritional education within school curricula for children but also involve parents in nutrition classes and practical workshops to increase knowledge and awareness^(37)^. Studies have found that parents are very supportive of the inclusion of food and nutrition education in primary schools, alongside the opportunity to attend parent nutrition classes and workshops^(38)^. These findings align with both the COM-B and TDF components focused on knowledge and psychological capabilities, and healthy eating interventions would need to address these areas. Previous research has also found parents tend to be positive about practical sessions such as taste tests and educational workshops^(34)^, similar to our own findings of the need for practical sessions alongside practical advice on high-fibre food swaps to increase parental motivation and involvement.

Our findings indicated that parents considered several social factors could become barriers to increasing fibre intakes; amongst the most cited were the parent–child conflict regarding food choices. These themes relate closely to the behavioural regulation and psychological capabilities included in the COM-B and TDF. Parent–child conflict around food choices has been reported in previous qualitative studies of parents’ views, with parents of children who are overweight more likely to report a lack of tools to deal with this conflict^(39)^. Previous literature suggests that effective parental strategies to be encouraged in this age group include guidance and education to promote healthy eating and restrictive guidance and rulemaking for deterring unhealthy food choices^(40)^. This is reinforced by parents who suggest activities such as trying new foods, encouraging children to get involved in meal planning and shopping, gardening, and restricting ‘treats’ are effective strategies^(39)^.

The current study suggests that children perceive barriers to increasing fibre intakes at an individual level such as difficulty in changing their current tastes and eating behaviours at different times of the day. Individual factors such as the opportunity to participate in taste tests and the need for lots of variety were also identified as potential facilitators for changing current behaviours. This is consistent with research identifying child-focused, and to a lesser extent, community-focused barriers are often reported as important from a child’s perspective compared with family- or culture-focused barriers^(41)^.

### Strengths and limitations

There are several limitations to this study. Although response rates were relatively high, time constraints of participants meant that it was not possible to arrange focus group for parents, and we relied solely on interviews; focus groups may have revealed different issues and encouraged more open dialogue and discussion of shared experiences. Interviewer bias may also have been more pronounced in the parent interviews as interviewers may be more likely to adapt or change their questions depending on the responses they received from the smaller number of participants. The topic of the research will also be prone to social desirability bias, with participants viewing responses that suggest a healthy and informed eating environment for their children to be more socially desirable. However, the semi-structured format of the interviews, interviewer–interviewee rapport and the consideration of this bias in the formation of the open-ended questions will hopefully have limited this bias. Participants included a number of children and parents of Black and ethnic minority origins; however, parents who were not confident with the English language may not have felt comfortable attending. Future research will need to consider the best approach to engaging parents through the use of formal or informal translators (e.g. other parents, school staff and family members). There was also a relatively high proportion of girls who participated (70 %), and having more boys participate may have highlighted different issues. Strengths of the study included the novel focus on increasing fibre intakes, and that the topic guides and analysis were theoretically informed.

### Implications

The facilitators and acceptable intervention components raised by parents and children in this study provide valuable insights and practical guides for dietary intervention development for primary school-based programmes. For parents, these included providing simple, practical and culturally acceptable information on high-fibre foods that could be easily incorporated into the child’s current eating behaviours, a gradual introduction of new foods with goal setting to motivate and encourage the progress, involvement and support from both parents and also extended family members, and practical sessions to introduce foods (taste tests, free samples and vouchers) for both parents and children. These activities were seen as necessary to help parents successfully make changes at home to support school-based dietary interventions. Children were also very positive about practical sessions, including taste tests, games and quizzes to help motivate them, and they also discussed behavioural regulation strategies they may adopt to resist unhealthy foods. Similar to other research exploring effective components of nutrition interventions^(42)^, these activities should be considered core components to any dietary interventions and act as levers of behaviour change. The COM-B model and TDF components are also reflected in many of these activities as determinants of behaviour, including capability, opportunity, and motivation, and can be effectively mapped to existing behaviour change techniques^(43)^.

## Conclusions

The findings of this study identified the overwhelming interest and support parents have for school-based dietary interventions, and the lack of knowledge about dietary fibre highlights the urgent need for a focused intervention aiming to increase fibre intakes in children. Children were also very positive about taking part in school-based activities to try and change their dietary behaviour and improve their diet. Many of the barriers identified are consistent with previous research, and further valuable parent and child insights are reported to overcome these modifiable barriers and bring about positive dietary change. Future dietary interventions which aim to increase dietary fibre intakes in children should address the barriers identified, including actively encouraging parent involvement, practical and informative sessions and effective home–school interactions.

## Supporting information

Donin et al. supplementary material Donin et al. supplementary material

## Supplementary material

For supplementary material accompanying this paper visit https://doi.org/10.1017/S1368980024000089
