# Supplementary material for: Identifying barriers and facilitators to increase fibre intakes in UK primary school children and exploring the acceptability of intervention components: a UK qualitative study
Source: Public Health Nutr. 2024 Feb 1;27(1):e59. doi: 10.1017/S1368980024000089 (PMC10897578; doi:10.1017/S1368980024000089)
Supplement: Donin et al. supplementary material [file S1368980024000089sup001.docx]

**Supplementary Table 1: PARENT FOCUS GROUP TOPIC GUIDE**

Aim: identify potential barriers and facilitators to uptake and maintenance of an intervention to increase fibre intakes in primary school children by replacing low fibre foods with high fibre alternatives during lunchtime and afterschool snack time.

| **Target behaviour – parental influence** |  | **Interview questions and prompts** |
| --- | --- | --- |
| Who needs to perform the behaviour? | Parents/carers |  |
| What do they need to do differently to achieve the desired change? | **Replace low fibre food items with high fibre foods for child in the packed lunch**  This would include changing bread type and replacing food items (low fibre, high sugar/fat items) with high fibre foods (high fibre breads, whole fruit/vegetables, high fibre crackers)  **Replace low fibre food items given after school for high fibre food items**  This will include not giving low fibre snacks (which are often high sugar/high fat foods) and replacing these with high fibre (lower sugar/fat) items such as whole fruits, high fibre snack bars, wholegrain crackers. | We are planning a study to see whether children are able to increase their fibre intakes by making a few changes to the foods they usually eat during the day. We have found that if children are given high fibre breakfast cereals to replace their usual low fibre cereal they are happy to eat this healthier option for a one-month period. We now want to see if children would be happy to do increase their fibre at other times of the day (swapping low fibre foods for higher fibre options (such as wholemeal breads, crackers, fruits and vegetables).  As a parent, how would you feel if you were asked to try giving your child different foods in their packed lunch to try and increase their fibre intakes? (such as a different type of bread, different snacks, fruit and vegetables).  If your child has school dinners, how would you feel if your child was asked/encouraged to eat the high fibre food options when possible?  In your home, who decides what goes in your child’s lunchbox?  *Prompt: are there many discussions/disagreements about lunch content?*  *How much choice does your child/children have in what goes in their lunch box?*  How do you think your child/children would feel about trying different/high fibre foods?  Would you have any concerns about asking your child to try different/high fibre foods?  *Prompt:*  *Do you think your child would be happy to try different foods?*  *If no -* Why wouldn’t they be happy?  Would you be more willing to try and include the foods if you knew your child had already tried them and rated them as ‘good’ or ‘very good’ in a taste test?  Would you be more willing if you were also given an initial free supply of the foods that you were asked to include?  Many children are given an after-school snack and these can often be low in fibre (and high in sugar/fat), therefore this is a good food item to target/swap to increase fibre intakes.  How would you feel about being asked to give your child different foods at snack time to try and increase their fibre intakes? (these may include high fibre snack bars, whole fruits, wholegrain crackers/biscuits)  How do you think your child/children would feel about trying different/healthier foods as snacks after school?  Do you think your child would be happy to try different snacks after school?  *If no -* Why wouldn’t they be happy? |
| When do they need to do it? | When the parent prepares the packed lunch and gives the after-school snack | Would the day of the week affect your ability to change your child’s food for high fibre items?  *Prompt: would afterschool activities affect what foods are eaten? Would your shopping habits (day of week when do shopping) alter what food items may be given (either in packed lunches or snacks).*  Does your child tend to have different snacks and lunches at weekends compared with week days?  *If yes* – what different types of food do they eat at weekends (find out if it is more fibre, less fibre or about the same).  Do they eat foods at different times at the weekend compared with weekdays?  *If yes* – How does it change at weekends (e.g., do they snack more regularly at weekends). |
| Where do they need to do it? | At home (food preparation) and possibly on the way back from school | Do you think there are situations that would make it difficult to replace after school snacks?  *Prompts:* Such as walking past corner shop on way home from school or visiting other’s houses, social/religious activities, swapping foods with other children, or habit of having sweet foods as a treat on certain occasions (e.g., after sport). |
| How often do they need to do it? | Week days for 6 months | We are planning to ask parents to encourage their children to replace foods with high fibre items every week day for 6 months. This will allow enough time to hopefully change food preferences of the children and improve the health of children.  How would you feel being asked if your child would like to participate in a study like this for 6 months?  *Prompt*: what concerns might you have or what problems might arise taking part for this length of time?  How does the food they usually eat change in school holidays*?*  Might this present any challenges for encouraging them to eat healthy/high fibre foods? |
| With who do they need to do it? | children | Do you think your child would enjoy taking part in a study like this? What do you think might make your child enjoy taking part/more?  Prompt: How might it affect them if children they know are taking part or if you join in by eating more healthy/high fibre foods? |

| **Capability-**  **Opportunity-**  **Motivation-**  **Behaviour (COM-B) component** | **Theoretical Domains Framework** | **What, potentially, needs to happen for dietary habits to change (low fibre foods to be replaced with high fibre items)?** | **Interview questions and prompts** |
| --- | --- | --- | --- |
| Physical capability | Physical skills | Availability of foods/buy high fibre food items? | When you are food shopping what tends to influence what you buy (thinking specifically about foods for your child’s lunches and snacks)  *Prompts: your child’s preferences, healthy choices/nutritional content, food costs (particularly current rising costs), new products, ease of preparation?*  Would you feel confident about buying foods which were high in fibre?  Prompts: Are you aware of which foods are high fibre? Are the labels on the back easy to follow? Would it help to have lists of high fibre foods? *If yes*: would you prefer this on a leaflet or as an app or on a website?  Do you think being provided with information about lunch and snack ideas to increase fibre intakes would be useful/make it easier i) to buy affordable high fibre items? ii) give your child high fibre items at lunch and snack time? If yes - would you prefer this on a leaflet/booklet or as an app or on a website? |
| Psychological capability | Knowledge | Need knowledge about fibre content of food items  Need knowledge about benefits of increasing fibre intakes?  Need awareness of usual (low) intakes of fibre in general population including children and children of different ethnic origins and risks to health  Need awareness of obesity crisis in primary school children and increasing fibre associated with healthy weight maintenance  Own dietary choices influence what child eats? | In the UK, in general we do not eat enough fibre in our diets, this is the case in all age groups, particularly children, and people of all ethnic backgrounds. Diets which are low in fibre are also known to be a risk factor for lots of chronic diseases such as type 2 diabetes, cancers and CVD. They are also associated with increased risk of obesity.  Where you aware that children are, in general, not eating enough fibre?  *Prompt: Does it surprise you? Would this information make you think about increasing your own child’s fibre intakes?*  Do you think children generally have healthy diets? Are you concerned about the effect children’s diets may have on their long-term health?  *Prompt: would you find being given more information on this useful if you were asked to try and change your child’s diet as part of a study?*  Do you think there would be any challenges from other family members if your child was asked to take part in a study which involved changing their breakfast, lunch and snack dietary habits?  *Prompt: do you think your own (or other family member’s) food preferences would influence what you would feel happy to give your child?*  Do you have any concerns around food allergies? If you do, would these affect your decision to let your child take part in a study like this one? What might help overcome those worries?  As an important component to this study, we would like to ask parents to support and encourage their child to eat the high fibre food items which would be either provided or recommended.  How would you feel about being asked to be part of the study with your child?  What information do you think would encourage parents to get involved? |
|  | Cognitive and interpersonal skills | Need to use encouraging language to help child eat the high fibre foods  Need to be able to encourage support from other family/carers who influence child’s dietary choices/intakes |  |
|  | Memory, attention and decision processes | Need to remember to buy the high fibre foods  Need to decide that buying these items and including them in child’s diet is an important thing to do  If they have other children, need to consider if they would make changes to all children’s diets  Need to consider influences of their own childhood diets  Need to consider influence of marketing aimed at children and consumption of high fat/sugar foods | If your child was asked to take part in a study like this one, how would you feel about changing your shopping habits – would you mind being asked to buy different food items if they were a similar cost?  Prompt: Might you need to go to different shops?  There are lots of adverts for sweets and cakes and sugary foods which are aimed at children, do you find these adverts influence what your child finds acceptable to eat?  *Prompt: do you struggle to persuade your child to eat healthier choices of foods (for example biscuits, breakfast cereal, bread type) because they have seen adverts/posters for unhealthy foods?* |
|  | Behavioural regulation | Need to be able to encourage child to eat healthier options (and possibly abstain from high fat/sugar) alternatives  Need to use encouraging language with a flexible approach and not make discussions about dietary changes ‘argumentative’ | It can be very difficult to change what we eat, and this is certainly the case for children once they have developed their food preferences. We know that if children are given a food to try and asked to eat this for a short period they are happy to do this.  What do you think would be some good ways to encourage the child to **continue** to eat the high fibre food (i.e. for a 6-month period)?  As a parent, what might be the challenges you think you may face encouraging your child to continue?  As a parent, what do you feel might help you encourage your child to continue?  *Prompt: would examples of helpful responses to typical negative comments from children be useful? Goal setting with rewards (interactive charts/stickers/online games/app)? Regular contact with the research group (text message suggestions/events/monthly meetings)* |
| Social opportunity | Social influences | Need to be comfortable with who is delivering the intervention  Need for all of family/carers to support the dietary changes  Need to feel comfortable about discussing challenges with others | Does your child regularly eat an after-school snack? If yes, where would this be and who decides what the snack will be?  Prompt: does anyone besides yourself or your partner, or your child, choose which foods they have (e.g., child carer, other family members, their friends’ parents?)  If yes: How would you feel about explaining to them that your child is having healthy/high fibre foods?  If you have more than one child, would this be an issue?  *Prompt: would it be better to involve siblings in the study or might you consider changing sibling(s) lunch and snack time foods as well anyway? If yes, what might help to encourage siblings to change the foods they eat?*  What sort of characteristics of the research team (who would be offering dietary information and advice) would be important to you?  *Prompt: Nutritional qualifications, research experience, ethnicity (or knowledge of different cultural dietary practises), age of researcher, parental experience?*  Would you find it useful to have an informal parent group who you could share experiences/ideas/offer support whilst your child was taking part in the study?  If yes, how often would you think the group should meet – monthly? and with the research team? How would you prefer this group to meet (e.g., online, face to face)? |
| Reflective motivation | Professional/social role and identity |  | Would you say your culture, religion or background influences the dietary choices you make for your child?  *Prompt: would these make it harder to make dietary changes?*  Do you think your own memories of your childhood diet may influence what you give your child at lunch or snack time?  *Prompt: what might help reduce these influences if they are negative?* |
|  | Beliefs about capabilities | Need to feel confident their child will be happy to take part and change/maintain dietary foods  Need to feel confident others will support changes they make to child’s food  Need to believe 6 months timescale is appropriate study length | Do you think you would have support (from family or friends) if you wanted to make changes to improve your child’s diet?  The study would need to run for quite a long time (6 months), this is for several reasons, to help change child’s food tastes (takes time to do this), to help form healthy food habits, and to show a positive change in health (such as healthy weight maintenance, blood sugars).  How would you feel about being asked to be part of a study for this length of time?  *Prompt:* *would it be better to know why the study was needed for this length of time?*  How do you think your child would feel about being asked to be part of a study for this length of time? What do you feel would help your child want to continue with the study and the dietary changes? |
|  | Optimism | Need to be optimistic about need for research  Need not be pessimistic that an unhealthy, low fibre diet is inevitable for child | Do you believe that children’s diets are important for their health? Do you think that research into how to improve children’s diets is important?  Do you think it is possible to improve children’s diets? |
|  | Beliefs about consequences | Need to believe that dietary changes will be beneficial for child  Need to believe that being part of the study is something their child will benefit from | Do you think that making the changes to a children’s diet (like increasing high fibre foods at breakfast, lunch and snack time) would benefit your child’s health?  Do you think your child would benefit from being part of a study like this one?  *Prompt: would it be useful to hear about benefits of being part of the study for the child? (including other benefits such as learning about healthy diets, importance of fibre in foods, learning about scientific studies)* |
|  | Intentions | Need to have strong commitment to make the dietary changes for their child  Learn more about the how they can positively influence child’s diets to increase fibre intakes | If your child was asked to take part in this study, what might help you as a parent to commit to making the changes to the foods your child is offered at lunch and snack times?  Information about the health benefits?  Information about the high fibre options to buy?  Examples of lunch and snack foods to include?  Vouchers for high fibre foods to try?  Family events to encourage dietary changes?  Price comparisons of high and low fibre food items? |
|  | Goals | Need to understand and commit to the goals of the intervention  Need to be monitored by other people  Need to feel that goals are being monitored in an appropriate way  Need to feel that the goals are tailored to their needs  Need to aim to develop a routine that becomes habitual | Do you think having goals set during the study would help your child maintain their dietary changes? Such as monthly monitoring and rewards?  How would you feel about involving your child in agreeing the goals for changing what they eat?  What type of rewards might help your child to change what they eat?  Prompt: having lots of choice, having some days/snacks where they choose what they eat even if it’s low fibre, having other rewards.  Would you have any concerns around the checks or monitoring of progress for your child?  Do you think asking your child to complete a short dietary assessment every month would be acceptable way to monitor progress? As a parent, would you be happy to receive monthly text messages/questionnaires asking about your child’s progress? |
|  | Emotion | Need to feel positive/comfortable with dietary changes  Need to feel the dietary changes are not a threat to cultural identity  Need to feel comfortable about being in a control or treatment group | Would you feel comfortable letting your child take part in a study like this? Would you have any concerns about changing your child’s diet if this was focused on breakfast, lunch and snack time?  If your child was asked to take part, they may be part of a control group, which would mean they would not be asked to change the foods they eat or be given new foods to try, only asked to take part in the measurements (this may include blood samples and standard height and weight measures). As a parent would this be a concern for you? At the end of the six-month period, children who were part of the control group would then be given high fibre foods to try at home, information packs and vouchers thanking them for taking part – would you feel this was fair for those children who have not been part of the intervention? |

**Supplementary Table 2: CHILD FOCUS GROUP QUESTIONS**

Aim: identify potential barriers and facilitators to uptake and maintenance of an intervention to increase fibre intakes in primary school children by replacing low fibre foods with high fibre alternatives during lunchtime and afterschool snack time.

| **Target behaviour** |  | **Interview questions and prompts** |
| --- | --- | --- |
| Who needs to perform the behaviour? | Children | We are planning a research study to see whether children would be happy to change some of the things they eat at lunchtimes and after-school snack times. We want to find out how eating healthier, high fibre foods affect your health, so we would need to ask children to eat certain foods for about 6 months. How would you feel about being asked to take part in a study like that?  *Prompt: what might worry you about agreeing to take part? What would you like to know more about before agreeing to take part?* |
| What do they need to do differently to achieve the desired change? | Agree to and eat the high fibre food alternatives in their packed lunch  Agree to and eat the high fibre food alternatives for their afternoon snack | We would be focusing on changing lunches, and after school snacks. How would you feel about your parents/carers changing the foods they gave you for i) in your packed lunch, ii) after school snacks - for healthier/different foods?  What sorts of things might make you more likely to agree to changing what you eat at these times *(prompts: knowing it was better for you/liking the taste/rewards/friends also taking part?)* |
| When do they need to do it? | Lunch times and after school | Do you tend to have the same things in your packed lunches and snacks during the week?  Are there activities in the week (after school) which may make it difficult for you to change what you eat?  *Prompt: are there activities/times when you are given snacks by other people? If yes, would you be happy to have different snacks to others at these times?* |
| Where do they need to do it? | In school and after school (on way home, at home, other places) | How would you find changing what you eat at school? would you find it helpful/more comfortable if your friends were also being asked to eat particular foods? |
| How often do they need to do it? | Week days for 6 months | So that we can see how changing some foods might affect your health we would need to ask children to change what they eat for up to 6 months. How would you feel about changing what you eat at lunch times and snack times for that long? What might help to keep you motivated to eat the foods?  If you were part of a study like this, do you think you would be able to keep eating the healthier foods in the holidays? What might make it difficult to keep eating them / what would help to make it easier? (*prompts: extra rewards? Extra foods/choices given to take home?)* |
| With who do they need to do it? | Parents/carers, family | Do you think your parents/carers would be happy to give you different foods if you were asked to be part of a study like this?  If no, why do you think they wouldn’t be happy?  Are there other family members/people who look after you, who you think would not be happy for you to take part in a study like this?  If we asked your whole family to take part and make the same changes to what they ate, do you think you would find it easier to keep eating the healthier foods or would you prefer that you were the only one in your family switching to these foods? |

| **Capability-**  **Opportunity-**  **Motivation-**  **Behaviour (COM-B) component** | **Theoretical Domains Framework** | **What, potentially, needs to happen for dietary habits to change (low fibre foods to be replaced with high fibre items)?** | **Interview questions and prompts** |
| --- | --- | --- | --- |
| Psychological capability | Knowledge | Need knowledge about benefits of increasing fibre intakes?  Need knowledge of fibre contents in foods? | Do you think you have a healthy diet?  Would you like to have a healthier diet?  Do you feel you know how to eat healthily? Do you know which foods might be high in fibre? Do you know why we might want to eat more fibre?  Do you think if you learnt more about this you would be more willing to change what you eat so that you have more fibre in your diets? Do you think learning about this would be important to you to take part in this study? Do you think it might make you want to keep eating the high fibre foods? |
|  | Cognitive and interpersonal skills | Need to be able to have the language to communicate to family members/friends/carers about the study and ask for support when needed | If someone you knew thought it wasn’t a good idea to eat more healthier foods, how would that make you feel?  Do you think you would be happy to explain to them why you were taking part in a study like this?  Would it be useful if the study team talked about what you might say to people to help them understand?  If you had decided to take part in the study and try and eat the healthier foods but found it hard, who do you think might help you to eat the foods?  (*prompt: would it be useful to have your family involved/available to speak to, or teachers, or the research team or someone else?)* |
|  | Memory, attention and decision processes | Need to remember to eat the different foods  Need to decide that eating the high fibre foods was a good thing to do (and resist the sweeter options) | It can be very difficult sometimes to change what we eat and resist sweet snacks, as well as fatty snacks such as crisps. If you were asked to eat healthier snacks week days when do you think it would be the hardest to resist sweet/chocolatey/fatty snacks? What do you think might help you to resist?  *(prompt: walk a different route home, have healthier snacks with you, rewards)*  If you were asked to eat healthier foods during lunch and snack times, what might be some good ways to remind you to eat these foods?  *(Prompts: e.g., a sticker on your school bag, reminders from your family, preparing the food the day before, eating the same healthy foods every day, having a wall chart or card showing which foods you will eat each day,* *wall charts and stickers, special study lunch box, study website with activities, school reminders)* |
|  | Behavioural regulation | (linked to above?) | Who do you think would help encourage you to eat healthier foods and continue to take part in the study?  How will you manage if you have a strong craving for sweet or fatty foods?  (Prompts: would it help if your parents/carers were asked to remind you that you are not supposed to be eating these sweet, fatty snacks and to just let you have the healthier snack? – there are also ways we can beat those cravings e.g., remind themselves that the cravings will reduce soon after they start eating the healthy, distract themselves do you think it would help to learn bit more about beating cravings at the beginning of the study?) |
| Reflective motivation | Professional/social role and identity |  | There are children from lots of different backgrounds, cultures and religions in London and we want to make sure everyone feels happy to take part. Do you think there is anything in your own culture or religion which might make it difficult to change what you eat?  Do you think your family would be encouraging if you wanted to take part and eat the high fibre foods? |
|  | Beliefs about capabilities |  | Do you think you would be able to eat high fibre foods on weekdays if you were asked to take part? (if no, why not? if yes, why yes?). The study is for 6 months, do you think you would be able to continue to eat the high fibre foods for that long? What things might help you to carry on eating the foods?  Would it help to have lots of choice over which healthy foods you eat? Would it help to keep having taste tests in school so you can keep trying different foods? |
|  | Beliefs about consequences | Need to believe that dietary changes they were making were having a positive impact on health – in the long term? | Would it matter that you might not be able to tell you were healthier because of eating the healthier foods?  (because the changes you make now will keep you healthy for a really long time – would it be important to know that before you took part?) |
|  | Intentions | Strong commitment to maintain the changes (continue to eat the high fibre foods) |  |
|  | Goals | Need to understand and commit to the goals of the intervention  Need to be monitored by other people | If you were set goals through the study (for example, for the particular types of foods you will start eating, how close you were to eating the recommended amounts of fibre a day, how many portions of high fibre foods you managed to eat a day), and the team came back to the school regularly to see how you were getting on, would this help you to stick to the new foods? Would it help if you had rewards after each check-up if you had done well? What sort of rewards would motivate you and make you enjoy taking part?  Would you mind having the team checking to see how you were getting on? For example, by asking you to keep a diary of what you are eating. If yes, what would you not like about this?  If the study had a website you could visit with activities to do (such as quizzes or games around healthy eating), would that help you to stay motivated to eat the new foods? Would you be happy to use the website to send messages to the study team if you needed to? If no, would you prefer to speak with your teacher? |
| Automatic motivation | Reinforcement |  | What sorts of things might help motivate you to continue to eat the high fibre foods? And help make the foods part of your normal diet?  *parent/carer encouragement, learning more about health benefits, study pens/pads, group activities with other children taking part, vouchers at end of study, rewards of sweet treats at the weekend?)* |
|  | Emotion | Need to feel positive/comfortable with dietary changes | As we said, it can be really hard to change what we eat. If you took part in a study like this one, how do you think you might feel at the end if you had managed to eat all the new foods and started to eat more healthily?  How would you cope if you were feeling fed up because you were missing some of the sweet/fatty foods you used to eat?  Would it help much if you found you were feeling healthier and happier for eating the healthier foods? |

**Supplementary table 3**: Identified themes which relate to barriers and facilitators, mapped to the COM-B and TDF theoretical models, and recommendations for intervention design.

| **Themes related to barriers and facilitators** | **Capability-**  **Opportunity-**  **Motivation-**  **Behaviour (COM-B) component** | **Theoretical Domains Framework** | **Summary findings** | **Recommendations** |
| --- | --- | --- | --- | --- |
| Knowledge about dietary fibre and its health benefits | Psychological capability | Knowledge | Lack of parent knowledge of healthy eating and fibre in diet may act as a barrier to increasing fibre intake | Provide parents with information about the lack of fibre in diets, the importance of fibre to health, and how to identify high fibre food items/snacks. |
| Social factors  (parent/child conflict and choice, limited time, parental roles/conflicting attitudes, influence of wider family, peer influences) | Social opportunity | Social influences | Limited parental control over child’s food choices; limited time to make dietary changes; perception that family members may be unwilling to change their habits regarding unhealthy snacks for child; conflict between parents on use of treats or unhealthy snacks; children can be influenced by their peers to want unhealthy snacks. | Consider how to support parents to help their child make the dietary change; highlight the importance of both parents (and wider family) engaging where possible with the dietary change so they can support each other; consider techniques that will help children resist outside influences from other family members or friends; importance of group/peer activities to develop positive peer influence; acknowledge that children need to have choice and control when developing dietary change to improve diets. |
| Habit formation | Psychological capability | Behavioural regulation | Parents felt that child’s routine eating habits and behaviours may be difficult to change (e.g. being a ‘fussy eater’, liking unhealthy snacks, unwillingness to try new foods). Similarly, some children said it would be challenging to switch from favoured snacks. | Identify strategies that encourage children to try the new lunch/snack items (taste tests, free samples, activities to maintain engagement, giving them ownership by allowing choice over the items/snacks); support for parents to help them encourage their child; and the importance of the school setting for dietary interventions (having the message coming from school with reinforcement from parents) |
| Environmental Factors | Physical Opportunity | Environmental context and resources | Having the intervention within the school setting is viewed as helping to encourage children to make the dietary change | Findings reinforces the importance of the intervention being led from schools; parents consistently report children respond well to messaging on dietary changes when led by school and more likely to be open to behaviour changes; reinforces the importance of engaging activities such as taste tests within the school setting to introduce and reinforce behaviour changes. |
| Choice and the need for variety | Reflective motivation | Beliefs about capabilities, empowerment | Children felt strongly about the need for lots of choice and variety when making dietary changes to overcome concerns that they would dislike all high fibre foods. | Build in some element of choice over lunch/snack items; strengthen self-efficacy and resilience to empower children to make and sustain dietary behaviour changes. |

**Supplementary table 4:** Identified themes which relate to intervention characteristics mapped to the COM-B and TDF theoretical models, and recommendations for intervention design.

| **Themes related to acceptable intervention characteristics** | **Capability-**  **Opportunity-**  **Motivation-**  **Behaviour (COM-B) component** | **Theoretical Domains Framework** | **Summary findings** | **Recommendations** |
| --- | --- | --- | --- | --- |
| Introduction to and rational for the intervention | Psychological capability | Knowledge, beliefs about consequences | Information about the rational for an intervention to increase fibre intakes, the health benefits of increasing fibre intakes, and information and examples of high fibre foods they could try. Most children felt that having more information about the benefits of eating more fibre would encourage them to make dietary changes. | Ensure clear information about the dietary intervention so that parents understand the aims and importance; provide parents (and children) with clear information about the importance of fibre in diet to raise their own awareness and to support them when talking to their children about why they should be increasing the fibre in their diets, the information should include examples of high fibre lunch and snack items parents can buy. |
| Tailoring of an intervention | Reflective motivation | Social role and identity | Parents want information that takes account of their cultural/ethnic identity | Take account of cultural and ethnic diversity in local areas, including dietary traditions as well as language needs. Intervention materials should be translated where necessary. |
|  | Psychological capability | Behavioural regulation | Starting off with small, manageable steps may be easier for children/parents to make the dietary changes. | Consider what is a realistic and manageable first step for children and their parents to take in any dietary intervention, and how to pace this. |
|  | Automatic motivation | Reinforcement | Parents were happy to receive reminders and further information on increasing fibre, children also wanted to have feedback and monitoring to help motivate them to sustain changes. | Regular communication may include texts or emails to remind parents about the intervention and to monitor progress, important to consider reasonable level of frequency for emails/texts to ensure parents are not over-burdened. Consideration should also be given to how parents can reply or provide feedback. Feedback to the children on how they are doing may help motivate them to continue. |
| Social support | Psychological capability | Cognitive and interpersonal skills | All parents were keen to support their child make dietary behaviour changes. Children also agreed that their parents would offer them the most support to make the changes to their diet. Some parents suggested that other parents would be a positive source of support to help them make changes to their child’s diets | Consider the provision of a parent support forum to chat about issues they encounter and support each other in helping their children engage with the study. |
| Behavioural regulation skills | Reflective motivation | Beliefs about capabilities, intentions | Children identified several techniques they would use if they were trying to resist unhealthy snacks and sustain any dietary behaviour changes they had made. | Include promoting the use of techniques to help the children regulate their behaviour to achieve the dietary behaviour changes they would like. |
| Practical aspects of an intervention | Physical opportunity | Environmental context and resources | Parents wanted a simple and focused approach involving small changes that they can easily make. They also want information that is clear and easily accessible. Parents will be encouraged to buy HF items if they are affordable, available in their usual retail outlets and if they receive vouchers and free samples to try. | Consider presentation of information – clear, easy for parents to absorb, possibly using visual aids such as graphics, provide parents with samples for them to try at home with their children, and a voucher to buy the high fibre items. Need to source high fibre lunch/snack items that are readily available in mainstream food outlets including supermarkets (in particular, those outlets considered to be more affordable). |
| Modes of intervention delivery | Reflective motivation | Goals. | Parent and child data indicated that it was important to provide reminders to parents/children, encourage them to monitor progress against goals, provide activities to keep children engaged in the behaviour change, and that they find interesting and fun | consider a range of engagement activities to suit different preferences; factor in some that will involve children in some way including making things, games, group activities. Setting goals and rewarding the children will be important to encourage them to maintain the behaviour change. |
